# The Sufferings of Christ

**Published:** 1887-12-10

**Authors:** 


### Words of Consolation.

[Communications for this column should be addressed "Chaplain," care of the Editor of The Hospital, who will gratefully welcome any suggestions or inquiries from matrons, nurses, and the staff generally.]

### LXI.—The Sufferings of Christ.

*(For Reading to the Sick.)*

"They stripped him." Here is another detail of the Passion which will bear looking into by the light of other passages of Scripture. Could He have identified Himself more closely than this with our fallen humanity? Oh! what must He not have endured, with all His exquisite sense of dignity, delicacy, and refinement, to have been treated with such vile rudeness by creatures lost, it would seem, to all sense of shame. "He was put to an open shame" (Heb. vi. 6). Though he knew no sin and had nothing in Him to be ashamed of, yet He was put to shame. At no time could His sweet refinement, and (we say it with reverence) His truly Divine sense of propriety, have been more shocked than when His persecutors stripped Him of His raiment previous to putting on the purple robe, and left Him naked and bleeding. You remember how closely connected were the nakedness and the shame in the Garden of Eden when once sin had done its deadly work. The Lord God called unto Adam, "Where art thou?" The answer was, "I was afraid, because I was naked, and I hid myself." And because the man and woman felt the sense of shame so deeply, of which they had been quite ignorant in the period of their innocence, they made themselves aprons of fig leaves and clothed themselves with the skins of animals. In order that we might regain our lost innocence we find our Lord submitting to this depth of degradation, as the Sin-bearer, enduring the burden of shame that He might clothe us in the garments of righteousness. What then shall we think of shame? We know well enough what it is to feel ashamed; and we know how this sense of shame revives again and again with fearful accuracy as remorse, or a better sorrow, succeeds the excitement of any indulgence in sin. There is a use to be made even of shame. But this feeling must be taken advantage of speedily, or shame will lose its intended power for good, and sin be continued without shame. St. Paul even speaks of some "who glory in their shame" (Phil. iii. 19). Shame is, after all, a very merciful provision, given in order that we may feel our nakedness, and desire "the fine inen which is the righteousness of the saints." But if lour sense of shame is ever to be used as a first stepping-stone to such a blessed result, we must guard most zealously against the temptation which always comes with it, to dissemble, cloak, or it may be to hide sin altogether—anything sooner than confess it. The outer and visible glory of heaven will be the raiment white and glistening; the body raised in corruption will be transfigured even as our Lord's; but this will be the result of many a humiliation here in the struggle to regain the garments of innocence and purity.

